# Mmu-miR-27a-5p-Dependent Upregulation of MCPIP1 Inhibits the Inflammatory Response in LPS-Induced RAW264.7 Macrophage Cells

**DOI:** 10.1155/2015/607692

**Published:** 2015-07-30

**Authors:** Ying Cheng, Li Du, Hanwei Jiao, Huapei Zhu, Kailian Xu, Shiyu Guo, Qiaoyun Shi, Tianjing Zhao, Feng Pang, Xiaoxiao Jia, Fengyang Wang

**Affiliations:** College of Agriculture, Hainan University, Hainan Key Lab of Tropical Animal Reproduction & Breeding and Epidemic Disease Research, Animal Genetic Engineering Key Lab of Haikou, Haidian Island, Haikou 570228, China

## Abstract

Lipopolysaccharide (LPS) stimulates macrophages to release proinflammatory cytokines. MicroRNAs (miRNAs) are short noncoding RNAs that are involved in inflammatory reaction. Our previously study identified the downregulated expression of mmu-miR-27a-5p in RAW267.4 cells treated with LPS. To dissect the mechanism that mmu-miR-27a-5p regulates target genes and affects proinflammatory cytokine secretion more clearly, based on previous bioinformatics prediction data, one of the potential target genes, MCPIP1 was observed to be upregulated with qRT-PCR and western blot. Luciferase reporter assays were performed to further confirm *in silico* prediction and determine that MCPIP1 is the target of mmu-miR-27-5p. The results suggested that mmu-miR-27a-5p directly targeted the 3′-UTR of MCPIP1 and the interaction between mmu-miR-27-5p and the 3′-UTR of MCPIP1 is sequence-specific. MCPIP1 overexpression decreased the secretion of IL-6, IL-1*β*, and IL-10 in macrophage cells stimulated with LPS. Our findings may provide the important information for the precise roles of mmu-miR-27a-5p in the macrophage inflammatory response to LPS stimulation in the future.

## 1. Introduction

Innate immunity plays important role in defense against pathogens, and lipopolysaccharide (LPS) triggers innate immunity [1, 2]. LPS stimulation activates multiple signaling pathways in macrophages, which results in production of inflammatory cytokines, regulation of adhesion molecule expression, and induction of inducible nitric oxide synthase (iNOS) expression [3–5]. These activities are essential* in vivo* in controlling and maintaining the balanced inflammatory reactions.

MicroRNAs (miRNAs) are about 22 nt endogenous RNAs that can play important gene regulatory roles in specifying mRNA cleavage or repression of productive translation [6, 7]. Accumulating evidences suggested that miRNAs were involved in innate immunity. LPS stimulation upregulates or represses the expression of miRNAs in macrophages. MiR-155 is upregulated in response to LPS, promoting the expression of TNF-*α* and destabilization of PU.1 [8–10]. MiR-155 also targets and suppresses SOCS1 in regulatory T cells [11, 12]. In contrast, miR-125b inhibits the expression of TNF-*α* and is downregulated in LPS-treated macrophages [9]. In response to stimulation by LPS or other microbial components, a rapid increase of the expression of let-7e, miR-146, miR-21, miR-155, and miR-181c has been observed in monocytic cell lines or mouse macrophages [8, 12–14]. MiR-21 negative regulates TLR4 signaling through targeting PDCD4 in human peripheral blood mononuclear cells stimulated with LPS [15]. With miRNAs array, more than 25 miRNAs were found to be differentially regulated in LPS-treated RAW264.7 cells compared to untreated cells, including the upregulation of miR-155, miR-132, miR-22, miR-342, and miR-146 genes and the downregulation of miR-696, miR-805, miR-706, miR-710, and miR-214 genes [16].

The study in our laboratory has identified the downregulated expression of mmu-miR-27a-5p in* E. coli *LPS-stimulated RAW264.7, and the target genes of mmu-miR-27a-5p were predicted. With gene ontology (GO) classification, 18 inflammation-related candidate target genes were obtained [17]. However, few of these targets and regulatory pathways have been experimentally validated, and the functional confirmation of them is still needed.

To dissect the regulatory mechanisms that LPS affects innate immunity of macrophage more clearly, based on previous finding that mmu-miR-27a-5p in LPS-induced RAW264.7 macrophage was downregulated, in the study, we, for the first time, identify MCP-1-induced protein 1 (MCPIP1, also known as ZC3H12A and Regnase-1), which was originally found in human monocytes after treatment with MCP-1 and is proapoptosis [18], as a target of mmu-miR-27a-5p. We also demonstrate that mmu-miR-27a-5p regulates the LPS-induced upregulation of MCPIP1, as well as subsequent proinflammatory cytokine secretion. Our studies provide conclusive mechanistic evidence for mmu-miR-27a-5p as regulator of MCPIP1 signaling events that play important roles during innate immune response.

## 2. Materials and Methods

### 2.1. Cell Lines and LPS Stimulation

RAW264.7 cells were obtained from the Cell Bank of the Chinese Academy of Science (Shanghai, China) and were routinely grown as previously described [19].* E. coli* LPS for stimulation was prepared as previously described [19].

### 2.2. qRT-PCR for miRNAs and mRNAs Expression

To determine the miRNA expression levels, small RNA was extracted from the stimulated RAW264.7 or unstimulated, and qRT-PCR was performed as previously described [17]. To analyze the levels of mRNAs from the stimulated RAW264.7 or unstimulated, the high-quality total RNA was isolated and qRT-PCR was performed as previously described [20].

### 2.3. Western Blot Analysis

The total proteins from stimulated RAW264.7 or unstimulated were harvested and western blots were performed as previously described [19]. Primary antibodies included goat polyclonal anti-MCPIP1 (1 : 200 dilution, Santa Cruz Biotechnology, USA), and GAPDH rabbit mAb (1 : 1000 dilution, Cell Signaling Technology, USA). Secondary antibodies used were horseradish peroxidase- (HRP-) labeled rabbit anti-goat IgG (1 : 4000 dilution, Santa Cruz Biotechnology, USA) and HRP-labeled goat anti-rabbit IgG (1 : 4000 dilution, Santa Cruz Biotechnology, USA).

### 2.4. MiR Mimics and Transfection of miRNAs

hsa- (mouse-) miR-27a-5p Pre-miR miRMA precursor (catalog number AM17100) and its scrambled control Pre-miR negative control number 2 (catalog number AM17111) were obtained from Ambion (Austin, TX, USA). For miRNA transfection studies, RAW264.7 cells were seeded in six-well plates and were transfected with 100 nM mmu-miR-27-5p or with a scrambled miRNA (SCR-miRNA, Ambion catalog number AM17110), using X-tremeGENE siRNA transfection reagent from Roche (Indianapolis, IN, USA) according to the manufacturer's protocol.

### 2.5. 3′-Untranslated Region (3′-UTR) Cloning and Mutation

The 3′-UTR sequence of MCPIP1 and 3′-UTR of GAPDH was amplified from mouse cDNAs by PCR, and the putative miRNA-binding site 5′-TAAGCCC-3′(478–484) in the 3′-UTR sequence of MCPIP1 was mutated to 5′-GTTTTTT-3′ by PCR. The primers were listed in Table 1(a). The wild-type and truncated mutation of 3′-UTRs of MCPIP1 were amplified by PCR and the obtained fragments with Sac I and Sal I sites were cloned into pmirGLO Dual-Luciferase miRNA Target Expression Vector. The primers were listed in Table 1(b). The constructs were validated by sequencing.

### 2.6. Luciferase Assay

RAW264.7 cells were seeded into a 96-well plate. After 24 h, the cells were cotransfected with the wild-type and mutated MCPIP1-3′-UTR reporter plasmid and pmirGLO plasmids or transfected with Pre-miR-27-5p and miR-scrambled control precursors (miR-SCR). At 36 h after transfection, luciferase assays were performed using the Dual-Luciferase Reporter Assay System (Promega, WI, USA) according to the manufacturer's instruction. Unless otherwise specified, negative control and miRs were used at 60 nM concentration.

### 2.7. Mouse MCPIP1 cDNA Cloning, Expression Plasmids Constructing, and Cell Transfection

According to MCPIP1 sequence information (NM_153159), specific primes (forward: 5′-GGTGGCCTCGAGGCCACCATGAGTGACCCTTGTGGAAC-3′; reverse: 5′-ATTATTAAGCTTCTCACTGAGGTGCTGGGA-3′) were designed and mouse MCPIP1 full length cDNA was cloned by RT-PCR. The mouse MCPIP1 full length cDNA was inserted into pEGFP-N1 (Clontech) and the recombinant plasmid pmMCPIP1-EGFP was constructed. After validating by sequencing, pmMCPIP1-EGFP or pEGFP-N1 was transfected into RAW264.7 cells by electroporation. Briefly, RAW264.7 cells were pelleted and resuspended (2 × 10^7^ cells/mL). Then, the electroporation was performed using a ECM830 (BTX, Holliston, MA) (175 V, 90 msec, 1 pulse), 10 *μ*g pmMCPIP1-EGFP, or pEGFP-N1 plasmid was electroporated into 500 *μ*L cell suspension, respectively. The electroporated cells were resuspended in 5 mL complete medium (10% FBS) and seeded into 12-well plates in 5% CO_2_ at 37°C for the indicated times (Figure 3(b)).

### 2.8. Cytokine ELISA

At 1 h after electroporation, the supernatant and unadherent cell were washed away carefully, and the adherent cells were incubated in fresh complete medium supplemented with 1 *μ*g/mL LPS for 3, 6, 12, 24, and 48 h, respectively. Then, the supernatant were collected for analyzing the secretion of IL-6, IL-1*β*, and IL-10 release using enzyme-linked immunosorbent assay (ELISA) kits (Figure 3(b)), Mouse IL-6 ELISA Kit (Boster, China), Mouse IL-1*β* ELISA Kit, and Mouse IL-10 ELISA Kit (ExCell Biology, China). According to the manufacturer's instructions, the assays were performed using a Model 680 Microplate Reader.

### 2.9. Statistics

Student's *t*-test and one-way ANOVA were performed for significance analysis as previously described [19]. A *P* value of <0.05 (^*∗*^) was considered significantly different and a *P* value of <0.01 (^*∗∗*^) was considered extremely significant.

## 3. Results and Discussion

### 3.1. Expression of the Potential Target of Mmu-miR-27a-5p, MCPIP1, Is Upregulated in Macrophage Stimulated with LPS

**Figure 1:**
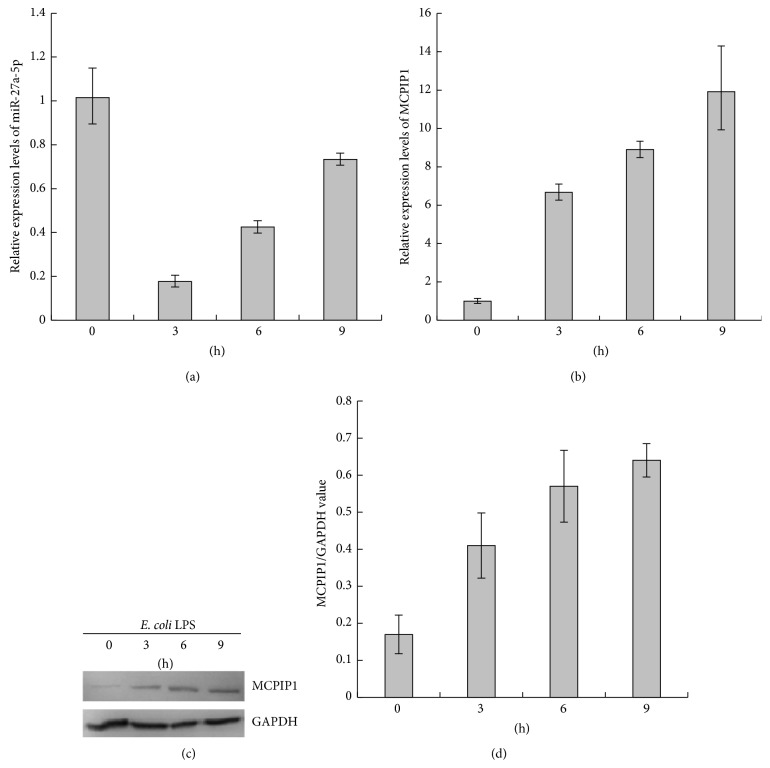
The potential target gene of mmu-miR-27a-5p, MCPIP1, is upregulated in macrophage stimulated with LPS. (a) LPS time-dependent mmu-miR-27a-5p expression. (b) Relative expression level of MCPIP1 mRNA under* E. coli* LPS stimulation for 6 h. (c) MCPIP1 protein expression under* E. coli* LPS stimulation. (d) Quantification of the western blots with BandScan 5.0 (*n* = 3).

As demonstrated in our previous report, miRNA profiling of LPS-treated RAW264.7 macrophage cells was performed by the initial high throughput array-based screen and further qRT-PCR validation, and the downregulation of mmu-miR-27a-5p was detected [17]. To dissect the expression trend of mmu-miR-27a-5p which is involved in the macrophage response to LPS stimulation more clearly, in this study, the LPS time-dependent mmu-miR-27a-5p expression was determined by qRT-RCR analysis. As shown in Figure 1(a), TaqMan MicroRNA Assay Kit (Applied Biosystems) based on stem-loop RT-PCR principle was used to determine the expression level of mmu-miR-27a-5p in RAW264.7 stimulated with LPS (3, 6, and 9 h), and the expression level of miR-27a-5p in RAW264.7 unstimulated with LPS was used as control (0 h, line 1). At 3 h after incubation with LPS (1 *μ*g/mL), mmu-miR-27a-5p was downregulated, and a gradual increase was observed from 3 h to 9 h; however, at 9 h after incubation with LPS (1 *μ*g/mL), the expression level of mmu-miR-27a-5p was still lower than that under no stimulation condition (0 h). The plausible mechanisms that mmu-miR-27a-5p is downregulated in RAW264.7 macrophages might be as follows. (1) Under the stimulation of LPS, the accumulation of pri-miRNA of mmu-miR-27a-5p is inhibited by the inhibiting of Pol II; (2) under the stimulation of LPS, Dicer is inhibited; the process that pri-miRNA of mmu-miR-27a-5p is cut into double stranded miRNA is inhibited.

**Figure 2:**
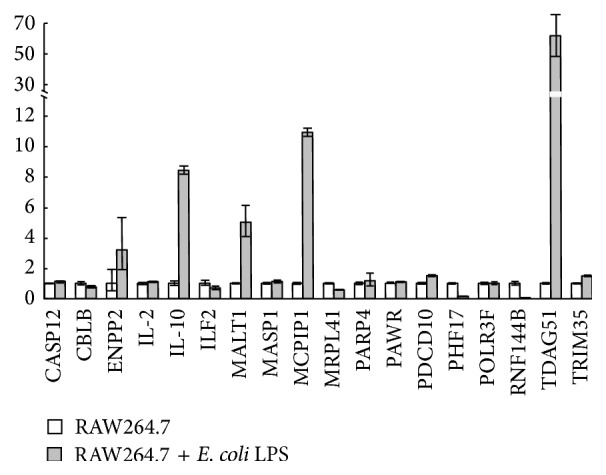
Relative mRNA expression level of eighteen candidate target genes of mmu-miR-27a-5p under* E. coli *LPS stimulation for 6 h.

In our previous report, with bioinformatics approaches and GO classification, 18 inflammation-related genes were predicted to be the potential targets of mmu-miR-27-5p [17]. Based on these findings, to further validate the predict results, the specific primers were designed for qRT-RCR, which were performed to analyze the mRNA expression levels of 18 inflammation-related genes (Table 2). As shown in Figures 1(b) and 2, the upregulation of MCPIP1 mRNA in RAW264.7 stimulated with LPS (1 *μ*g/mL) was observed.

To further confirm the upregulation of MCPIP1 expression, western blots were performed. After incubation with LPS (1 *μ*g/mL) for 0, 3, 6, and 9 h, respectively, the total proteins were extracted, and the protein expression levels of MCPIP1 were determined; the results indicated that the MCPIP1 protein expressions were markedly enhanced from 0 h to 9 h (Figures 1(c) and 1(d)).

### 3.2. Mmu-miR-27a-5p Targets MCPIP1

To further confirm* in silico* prediction and determine that MCPIP1 is the target of mmu-miR-27a-5p, luciferase reporter assays were performed. The 3′-UTR regions of mouse MCPIP1 (NM_153159) and mouse GAPDH (NM_001289726) were cloned, and then, which was cloned into pmirGLO-luciferase reporter plasmid, to generate pmirGLO-luciferase-MCPIP1-3′UTR-WT and pmirGLO-luciferase-GAPDH-3′UTR-WT, we transfected HEK293 cells with these constructs in the presence of mmu-miR-27a-5p. The results indicated that, compared with that of cotransfection with pmirGLO-luciferase-MCPIP1-3′UTR-WT and SCR-miRNA, the luciferase activity in HEK293 cells cotransfected with pmirGLO-luciferase-MCPIP1-3′UTR-WT and mmu-miR-27a-5p was reduced about 30%, thereby showing mmu-miR-27a-5p reduced luciferase activity via MCPIP13′-UTR.

**Figure 3:**
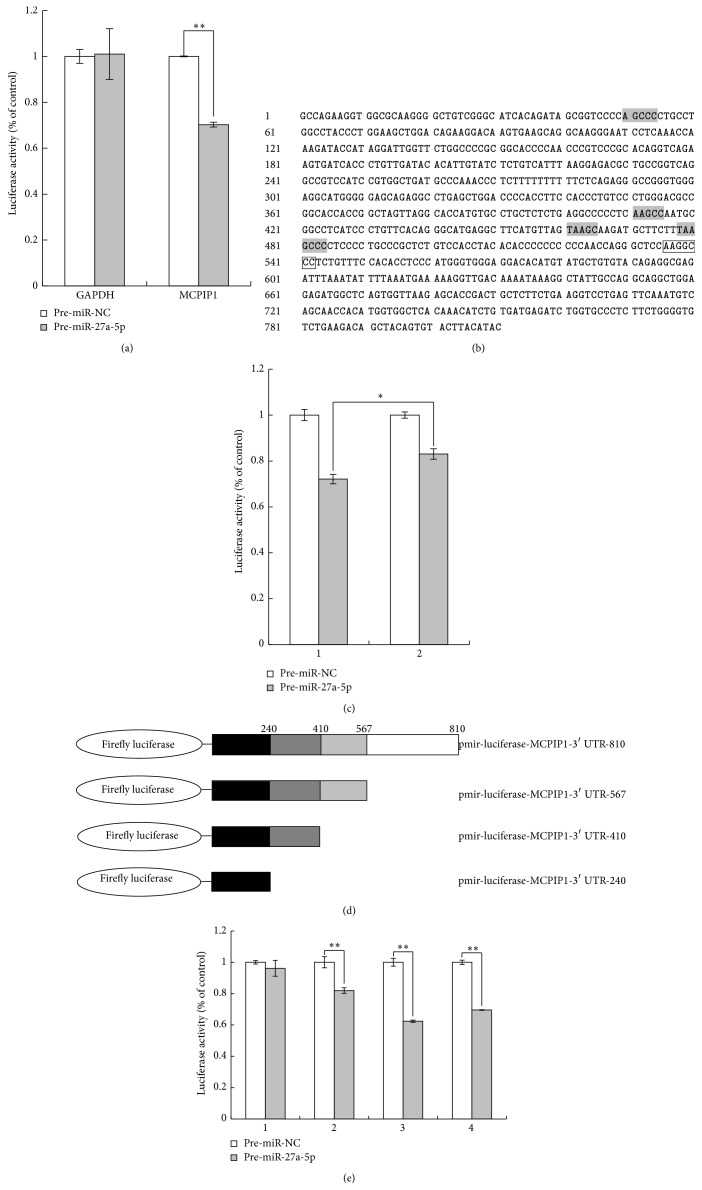
Mmu-miR-27a-5p targets MCPIP1. (a) RAW264.7 were transfected with a luciferase construct containing MCPIP1 3′-UTR (MCPIP1 -UTR-luc) and GAPDH 3′-UTR (GAPDH-UTR-luc) in the presence of mmu-miR-27a-5p. (b) Putative binding site of mmu-miR-27a-5p in MCPIP1 3′-UTR. (c) Firefly luciferase activity of the deletion mutant of predicted target sites. Firefly luciferase activity of HEK293 cells cotransfected with Pre-miR-NC (negative control, white bars) or Pre-miR-27a-5p (gray bars) plus pmir-luciferase-MCPIP1-3′-UTR (lane 1) and pmir-luciferase-MCPIP1-3′UTR-mut (lane 2), respectively. Results depict the average (±SEM) of three independent transfections. (d) A series of reporter plasmids was constructed for luciferase assays. (e) Firefly luciferase activity of HEK293 cells cotransfected with Pre-miR-NC (negative control, white bars) or Pre-miR-27a-5p (gray bars) plus pmir-luciferase-MCPIP1-3′UTR-240 (lane 1), pmir-luciferase-MCPIP1-3′UTR-410 (lane 2), pmir-luciferase-MCPIP1-3′UTR-567 (lane 3), and pmir-luciferase-MCPIP1-3′UTR-810 (lane 4).

Computational analysis results suggested that there is a putative binding site (478–484 bp from the start site of the 3′-UTR) of mmu-miR-27a-5p in MCPIP1 3′-UTR (Figure 3(b)). To determine whether mmu-miR-27a-5p binds to the sites and represses MCPIP1 expression, a firefly luciferase reporter vector was constructed by deleting the seed sequence in the 3′-UTR of MCPIP1. The plasmid was transfected into HEK293 cells together with either mmu-miR-27a-5p oligonucleotides or scrambled miRNA oligonucleotides. Interestingly, although not 100%, the deletion of the binding sites did partly abolish the activity of mmu-miR-27a-5p in repressing luciferase activities (Figure 3(c)), which suggested that mmu-miR-27a-5p downregulates the expression of MCPIP1 through direct interaction with the specific miRNA-binding sites in the 3′-UTR sequence of MCPIP1. Using bioinformatics analysis, several potential miRNA-binding sites in the 3′-UTR sequence of MCPIP1 were also predicted (Figure 3(b)), which can explain why the deletion of the binding sites did partly, not 100%, abolish the activity of mmu-miR-27a-5p in repressing luciferase activities.

To further investigate whether mmu-miR-27a-5p represses MCPIP1 expression through its binding to the putative binding site in the 3′-UTR of MCPIP1, we constructed three more luciferase plasmids, which contain the mutant 3′-UTR sequence with deletion of 568–810 fragment, 411–810 fragment, and 241–810 fragment, respectively (Figure 3(d)). These plasmids were transfected into HEK293 together with mmu-miR-27a-5p oligonucleotides. The deletion of 568–810 fragment did abolish the activity of mmu-miR-27a-5p in repressing luciferase activities about 40%; the deletion of 410–810 fragment did abolish the activity of mmu-miR-27a-5p in repressing luciferase activities nearly 20%; the deletion of 240–810 fragment did almost abolish the repression of mmu-miR-27a-5p (Figure 3(d)). Thus, these results support the* in silico* prediction that mmu-miR-27a-5p directly targeted the 3′-UTR of MCPIP1, which suggest that the interaction between mmu-miR-27a-5p and the binding sites in the 3′-UTR of MCPIP1 is sequence-specific; however, the information about the binding sites from bioinformatics software prediction is limited, which is majorly based on the primary structure of 3′UTR of MCPIP1, not the second structure.

According to the theoretical prediction, there should not be any significant changes between lanes 1 and 2 in Figure 3(e). However, in Figure 3(e), the deletion of 411–810 fragment did still abolish the activity of miR-27a-5p in repressing luciferase activities about 20% (lane 2), which is significantly different from that of Pre-miR-27a-5p plus pmir-luciferase-MCPIP1-3′UTR-240 group (lane 1). The reasons might be as follows. In 241–409 fragment of the 3′-UTR, there might be other potential binding sites of miR-27a-5p, which are not predicted by the bioinformatics analysis; the potential binding site (49–54 bp from the start site of the 3′UTR) is only the theoretical prediction, which cannot be verified by the firefly luciferase assay.

Additionally, the kinetics analysis of LPS-induced MCPIP1 expression indicated that a gradual increase of MCPIP1 was observed from 0 to 9 h (Figures 1(c) and 1(d)); however, the expression level of mmu-miR-27a-5p was still lower than that under no stimulation condition (0 h) (Figure 1(a)), which are also consistent with the fact that MCPIP1 is regulated by mmu-miR-27a-5p in RAW264.7 cells treated with LPS.

### 3.3. MCPIP1 Overexpression Decreased the Secretion of IL-6, IL-1*β*, and IL-10 in Macrophage Cells Stimulated with LPS

Toll-like receptors (TLRs) recognize microbial components and evoke inflammation and immune responses. As a TLR-inducible immune response modifier, MCPIP1 has an essential role in preventing immune disorders. Macrophages from MCPIP1(−/−) mice showed highly increased production of IL-6 and IL-12*β* in response to TLR ligands. By directly controlling the stability of a set of inflammatory genes including IL-6, IL-1*β*, IL-2, and IL-12*β*, MCPIP1 is an essential RNase that prevents immune disorders [21]. MCPIP1 promoter region (from −76 to +60) contains binding sites for the transcription factor Elk-1 and its partner SRF. In primary monocyte-derived macrophages, IL-1*β* rapidly induces the synthesis of MCPIP1, which takes place through the MAP kinase pathway and follows activation of Elk-1 [22].

**Figure 4:**
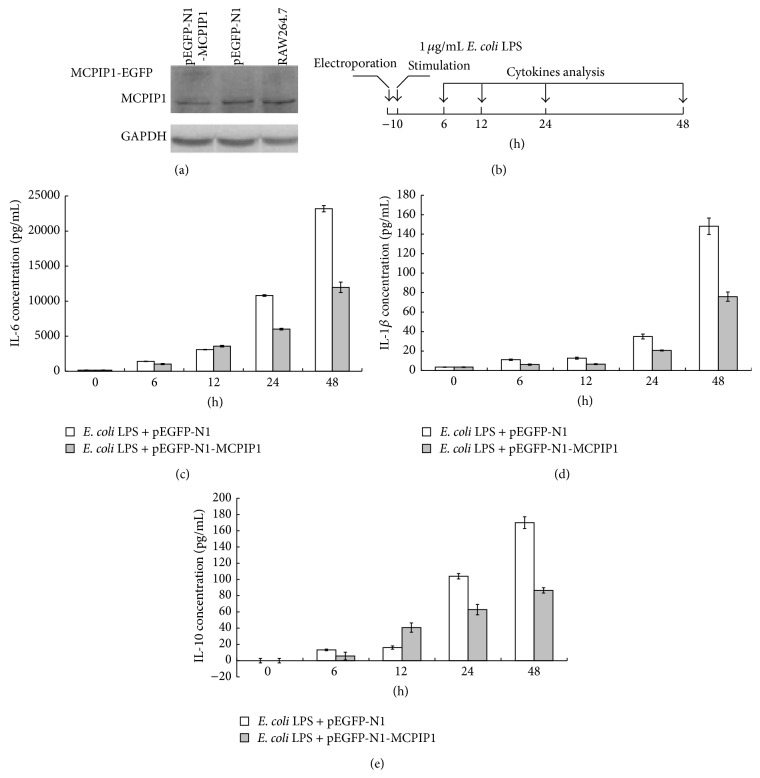
MCPIP1 overexpression decreased the secretion of IL-6, IL-1*β*, and IL-10 in macrophage cells stimulated with LPS. (a) MCPIP1 overexpression in macrophage cells stimulated with LPS. (b) Experimental design of cell stimulation experiments. (c) Secretion of IL-6 from RAW264.7 cells stimulated with* E. coli* LPS. (d) Secretion of IL-1*β* from RAW264.7 cells stimulated with* E. coli *LPS. (e) Secretion of IL-10 from RAW264.7 cells stimulated with* E. coli *LPS.

To determine whether the expression of MCPIP1 causes detectable dysregulation of cytokines known to be involved in inflammation reaction, MCPIP1 was overexpressed in RAW264.7 cells stimulated by* E. coli* LPS and the proinflammatory cytokine secretion, including TNF-*α*, IL-10, IL-6, IL-1*β*, IL-18, IFN-*γ*, and MIP1b, was determined. First, to confirm the expression of exogenous MCPIP1, pmMCPIP1-EGFP and pEGFP-N1 were electroporated into RAW264.7 cells, and at 6 h after electroporation western blot was performed on equal amounts of protein harvested from RAW264.7 cells electroporated with pmMCPIP1-EGFP, RAW264.7 cells electroporated with pEGFP-N1, and RAW264.7 cells. The results suggested that not only MCPIP1-EGFP fusion protein about 95 KD but also the endogenous MCPIP1 about 65 KD was detected with anti-MCPIP1 antibody (Figure 4(a)). Under fluorescence microscope, the fluorescence of the MCPIP1-EGFP fusion protein (at 6 h after electroporation) was also observed (data not shown).

After overexpressing MCPIP1, the secretion of IL-6, IL-1*β*, and IL-10, which were induced with* E. coli *LPS stimulation (48 h), was significantly reduced to approximately 50%, compared with that of RAW264.7 cells electroporated with pEGFP-N1 (Figures 4(c), 4(d), and 4(e)), and our findings that MCPIP1 overexpression decreases the secretion of IL-6 and IL-1*β* agree with that from previous reports [21, 23].

However, compared with that of control, the secretion of TNF-*α* in RAW264.7 simulated with LPS was not changed significantly. So, the data on expression levels of TNF-*α* were not shown in this report. Under the stimulation with LPS, the secretion of IL-18, IFN-*γ*, and MIP1b in RAW264.7 was not changed significantly. So, the data on the expression levels of IL-18, IFN-*γ*, and MIP1b were also not shown.

IL-10 is one kind of cytokine with two-way immune regulation. On the one hand, IL-10 inhibits the secretion of TNF-*α* and some other cytokines. On the other hand, IL-10 stimulates the proliferation and differentiation of B cells, the expression of MHC class II molecules, and activates immune system [24]. In RAW264.7 stimulated with* E. coli* LPS, the secretion of IL-1*β* and IL-6 was upregulated, which are necessary for the inflammation reaction; the secretion of IL-10 was downregulated slightly, which can negatively regulate the overproduction of IL-6 and keep the immune balance. Under the stimulation of LPS, the overexpression of MCPIP in RAW264.7 downregulates the secretion of IL-6, IL-1*β*, and IL-10 which can negatively regulate the overproduction of these cytokines, maintaining the balanced inflammatory reactions.

## 4. Conclusions

In summary, based on our previous study results, qRT-PCR was performed to validate the mRNA levels of 18 inflammation-related candidate target genes, including MCPIP1. We, for the first time, identify MCPIP1 as a target of mmu-miR-27a-5p by experimental validation and demonstrate that mmu-miR-27a-5p regulates the LPS-induced upregulation of MCPIP1. These findings suggested that mmu-miR-27a-5p might play an important role in* E. coli *LPS-induced immune response of macrophage via regulating the MCPIP1 expression, which maintains the balance of immune system of macrophage by regulating IL-6, IL-1*β*, and IL-10 secretion.

## Figures and Tables

**(a):** 

Number^*∗*^	Location	Sequence (5′-3′)
1	1–33	GGCGGAGCTCGCCAGAAGGTGGCGCAAGGGGCTGTCGGGCATC
2	796–766	GCGGTCGACCTGTAGCTGTCTTCAGACACCCCAGAAGAGG
3	1–19	GGCGGAGCTCGAAACCCTGGACCACCCAC
4	178–159	GCGGTCGACGGGTGCAGCGAACTTTATTG
5	451–511	TTCATGTTAGTAAGCAAGATGCTTCTTGTTTTTTCTCCCCTGCCCGCTCTGTCCACCTACA
6	511–451	TGTAGGTGGACAGAGCGGGCAGGGGAGAAAAAACAAGAAGCATCTTGCTTACTAACATGA

^*∗*^1: 3′-UTRMCPIP1-F; 2: 3′-UTRMCPIP1-R; 3: 3′-UTRGAPDH-F; 4: 3′-UTRGAPDH-R; 5: 3′-UTRMCPIP1-mF; 6: 3′-UTRMCPIP1-mR.

**(b):** 

Number^*∗*^	Location	Sequence (5′-3′)
7	1–23	GCGCGAGCTCGCCAGAAGGTGGCGCAAGGGGCT
8	810–776	GCCGTCGACGTATGTAAGTACACTGTAGCTGTCTTCAGACACC
9	567–543	GCCGTCGACCACCCATGGGAGGTGTGGAAACAGA
10	410–387	GCCGTCGACGAGGGGGCCTCAGAGAGCAGGCAC
11	240–213	GCCGTCGACCTGACCGGCAGCGTCTCCTTAAATGAC

^*∗*^7: 3′-UTRMCPIP1-F2; 8: 3′-UTRMCPIP1-R810; 9: 3′-UTRMCPIP1-R567; 10: 3′-UTRMCPIP1-R410; 11: 3′-UTRMCPIP1-R230.

**Table 2:** qRT-PCR primer for candidate target gene of mmu-miR-27a-5p.

Gene	GenBank ID	Primer sequence (5′-3′)
CASP12	NM_009808.4	F: CCAGATGCCCACTATTGAGAGAGT
		R: TGAGAGTTGCCTGTGCTAATTCC

CBLB	NM_001033238	F: TGTGGATGTTTCTTACCACTCGTT
		R: GTGATTATCCGAGGCATCTCTGT

ENPP2	NM_001285995	F: ACATGGTGCTGCCAGTTGAAT
		R: GACTGGGCACTGG0047AACCT

IL2	NM_008366.3	F: CCTGCAGGCATGTACAGCAT
		R: AAGGAGCACAAGTGTCAATGTGA

IL10	NM_010548.2	F: GATGCCCCAGGCAGAGAA
		R: CACCCAGGGAATTCAAATGC

ILF2	NM_026374	F: TTCTGGCTGCAGGACTGTTCT
		R: CACTCTCACACGGGTCAGTGA

MALT1	NM_172833.2	F: TTGCTTCCAAGCTTGTCGTATG
		R: CCGCTCCCGCTGAGGTA

MASP1	NM_008555	F: CCCAGAGGACAGGCACAATC
		R: GGAACATTCTGAGCTCTTGGGATA

MCPIP1	NM_153159.2	F: GCTGGCTGTGAACTGGTTTCT
		R: CAAGATGGCACAAACACGGTAA

MRPL41	NM_001031808.2	F: ATTTGTCGTCCCGGACTTGAC
		R: GCAGGAGCTCGGTAATTAACGT

PARP4	NM_001145978	F: TCCCTGGCTATGTCCATTGAG
		R: TCAGTTCATGTGTGTCACTGGAAA

PAWR	NM_054056.2	F: ATGCCATCACCCAGCAAAA
		R: CAGGTAGGATGTGCCTGGATCT

PDCD10	NM_019745.3	F: GCTGATGACGTAGAAGAGTACATGAT
		R: CCCGTGCCTTTTCATTTAGGT

PHF17	NM_001130184	F: GCGTTAGGCTACGTCGATATCC
		R: ACGCAGCATCCATATCATTGAG

POLR3F	NM_029763	F: TGTCTGAGCAGAAAGCCATCA
		R: TTTTCCAGGGCCGAACAG

RNF144B	NM_001170643.1	F: GACAATGACATATTCCTCAGGCACTA
		R: CTCGAGTGGCCGAGCTTATT

TDAG51	NM_009344.3	F: CAGATGGTGCAGTACAAAAATCG
		R: TGCTTCTGCCTGGTAGACTTGA

TRIM35	NM_029979.3	F: AGCTAGTGCTGGGTTGGAACA
		R: TCGATTCCGTGCCGAAAG
